# Cheiloplasty

**Published:** 1878-07

**Authors:** John H. Brinton

**Affiliations:** in the Jefferson Medical College Hospital


### ARTICLE IX.

### *Cheiloplasty.*

Surgical Clinic of John H. Brinton, M. D., in the Jefferson Medical College Hospital, May 29th, 1878.

Gentlemen.—The first case I shall bring before you is this little girl, six years of age, upon whom, five weeks ago, I performed a cheiloplastic operation, the formation of an entire upper lip. The original lip, you may remember, had been destroyed by sloughing following an attack of smallpox. Not only had the lip been destroyed, but also those parts of the superior maxillary bones which rest behind it and form its support. These had become necrosed, and came away in part, and were in part removed by Dr. Lamb, who had charge of the case at the time, and who has brought the child to this clinic for such help as surgery may give her. The operation I performed before you with the excellent advice and assistance of my colleague, Dr. Levis. I explained it to you at the time. You will recollect that large flaps were turned in from the cheeks, involving their entire thickness through into the mouth; that on the left side being the larger. These flaps were then united in the middle line by hare-lip pins, and the incisions on the cheeks were closed in the same manner, great care being taken to preserve the angle of the mouth.

You here see the result. The new upper lip is firm and strong, and all the incisions made by the operation have united. The success in this case is satisfactory, beyond my expectations; indeed, it is almost an exceptional result so far. You will notice that there is a little contraction of the newly placed tissues, with some narrowing of the mouth; this is unavoidable, and not to be wondered at when you reflect that all bony support is absent at the centre, owing to the deficiency of the maxillary bones. This contraction has occurred in spite of the very large size of the flaps. Where such large cicatrices are present, you must expect contraction, and you must remember that if you hope to attain success in plastic operations you must reach it by cutting large flaps and by free incisions.

Can I remedy this contraction? Yes, but not at this moment. I must wait until the contracting influence shall have spent itself, as it were, and until the new tissues shall have become old and solid. I shall then free the parts beneath, and perhaps do something more. In the meantime the parents of the child must be content, as, indeed, they are, with what has been already accomplished, and in the future I shall endeavor to perfect the result.—*Med. and Surg. Reporter.*

